# Assessing Sustainable Development Goal Target Indicator 3.5.2: Trends in alcohol per capita consumption in the Americas 1990–2016

**DOI:** 10.26633/RPSP.2021.142

**Published:** 2021-10-18

**Authors:** Maristela G. Monteiro, Camila B. Martins, Zila M. Sanchez, Jürgen Rehm, Kevin Shield, Rachael Falade, Jacqueline MacDiarmid, Pamela Trangenstein

**Affiliations:** 1 Pan American Health Organization Washington, D.C. United States of America Pan American Health Organization, Washington, D.C., United States of America; 2 Universidade Federal de São Paulo São Paulo Brazil Universidade Federal de São Paulo, São Paulo, Brazil; 3 Centre for Addiction and Mental Health Toronto Canada Centre for Addiction and Mental Health, Toronto, Canada; 4 University of Toronto Toronto Canada University of Toronto, Toronto, Canada; 5 University of North Carolina Chapel Hill, N.C. United States of America University of North Carolina, Chapel Hill, N.C., United States of America

**Keywords:** Alcohol drinking, alcoholism, sustainable development, public health, Americas, Consumo de bebidas alcohólicas, alcoholismo, desarrollo sostenible, salud pública, Américas, Consumo de bebidas alcoólicas, alcoolismo, desenvolvimento sustentável, saúde pública, América

## Abstract

The objective of this study was to estimate trends in alcohol per capita consumption from 1990 to 2016 in the Region of the Americas, covering 35 Member States. Data from the WHO Global Information System on Alcohol and Health were used to calculate the annual percent change of alcohol per capita consumption in each of the 35 countries of the Americas. The Americas as a whole showed no change in the total period, with a slight decrease in the period 2010–2016. From 1990 to 2016, all the countries that presented a trend of annual increase in annual percent change of alcohol per capita consumption were in the Caribbean and Central America. Large increases were found in the recent years in Cuba, Colombia, Uruguay, El Salvador, and several countries of the Non-Latin Caribbean. In conclusion, alcohol use remains a significant obstacle to the achievement of Sustainable Development Goal 3.5. To date, the policy response has been inadequate in protecting the people in the Americas from alcohol-attributable harms. Improving country capacity to collect and analyze data on alcohol per capita consumption is urgently needed to monitor progress on the Sustainable Development Goals and to serve to promote proven alcohol policies for reducing the harmful use of alcohol.

## RESUMEN

El objetivo de este estudio es estimar las tendencias en el consumo per cápita de alcohol desde 1990 hasta el 2016 en 35 Estados Miembros de la Región de las Américas. Se emplearon datos del Sistema Mundial de Información sobre el Alcohol y la Salud de la OMS para calcular la variación porcentual por año del consumo per cápita de alcohol en cada uno de los 35 países de la Región. En general, la Región no mostró cambio en todo el período, salvo una disminución leve entre el 2010 y el 2016. De 1990 al 2016, todos los países que registraron una tendencia al alza en la variación anual porcentual del consumo per cápita de alcohol se encontraban en el Caribe y Centroamérica. En los últimos años se observó un aumento importante en Cuba, Colombia, Uruguay, El Salvador y varios países del Caribe no latino. En conclusión, el consumo de alcohol sigue siendo un obstáculo significativo para lograr el Objetivo de Desarrollo Sostenible 3.5. Hasta la fecha, la respuesta de las políticas ha sido inadecuada para proteger a la población de la Región de los daños atribuibles al alcohol. Es necesario mejorar de manera urgente la capacidad a nivel de país para recopilar y analizar datos sobre el consumo per cápita de alcohol a fin de monitorear el progreso de los Objetivos de Desarrollo Sostenible y promover políticas relativas al alcohol cuya eficacia en cuanto a la reducción del consumo nocivo ha sido comprobada.

## RESUMO

O objetivo deste estudo foi estimar as tendências do consumo de álcool per capita de 1990 a 2016 na Região das Américas, cobrindo os 35 Estados Membros. Dados do Sistema Mundial de Informação sobre Álcool e Saúde da OMS foram usados para calcular a mudança percentual anual do consumo de álcool per capita de cada um dos 35 países das Américas. As Américas, como um todo, não mostraram mudança alguma no período total, com uma diminuição leve no período entre 2010 e 2016. De 1990 a 2016, todos os países que apresentaram uma tendência de aumento anual na mudança percentual anual do consumo de álcool per capita estão no Caribe e na América Central. Um grande aumento foi encontrado nos anos recentes em Cuba, Colômbia, Uruguai, El Salvador e vários países não latinos do Caribe. Em conclusão, o consumo de álcool continua sendo um obstáculo significativo para o cumprimento do Objetivo de Desenvolvimento Sustentável 3.5. Até o momento, a resposta política foi inadequada para proteger a população nas Américas dos danos atribuíveis ao álcool. Melhorar a capacidade dos países de coletar e analisar dados sobre o consumo de álcool per capita é urgentemente necessário para monitorar o progresso dos Objetivos de Desenvolvimento Sustentável e para promover políticas comprovadas de redução do consumo nocivo de álcool.

Alcohol consumption is a risk factor for population health, as it affects the risks of approximately 230 three-digit codes of the *International Statistical Classification of Diseases and Related Health Problems, 10th Revision* (ICD-10), including infectious diseases, noncommunicable diseases, and injuries (1). Alcohol is a psychoactive substance that impairs cognitive function acutely and chronically. It is toxic to cells and tissue functioning, it is carcinogenic, teratogenic (can alter fetal development at any stage of pregnancy), and due to its reinforcing properties, it can lead to dependence (2). Alcohol consumption continues to rank as a leading risk factor for the burden of disease worldwide and in the Americas (3).

Overall alcohol consumption is related to all-cause mortality and alcohol-specific mortality and disability (4, 5). Therefore, changes in consumption lead to changes in the overall as well as the alcohol-specific disease burden in a population. Mortality and morbidity are not the only burdens that alcohol creates; alcohol consumption also causes a large social burden (to society and the drinker’s social network) and economic burden (through costs such as lost productivity, health-care costs, and police, court, and prison costs) (6). Despite this, information on these burdens is not regularly collected (4–6). In 2016, alcohol caused at least 379 000 deaths and over 18 million lost disability adjusted life years (DALYs) in the Americas (3). Of all alcohol-attributable deaths globally, 52.4% are among those under 60 years of age (2).

The recognition of alcohol as a development issue in the Sustainable Development Agenda reflects its social, economic, and health impact (7). Within Sustainable Development Goal (SDG) 3 (Ensure healthy lives and promote well-being for all at all ages), SDG Target 3.5 aims to “strengthen the prevention and treatment of substance abuse, including narcotic drug abuse and harmful use of alcohol” (7). Two indicators to measure this target were adopted: 3.5.1 relates to the coverage of prevention and treatment of substance use disorders (including alcohol use disorders); and 3.5.2, specific to the harmful use of alcohol, is defined according to the national context as total alcohol per capita consumption (among those aged 15 years and older) within a calendar year in liters of pure alcohol (7).

The aim of this study was to analyze trends in alcohol per capita consumption (APC) from 1990 to 2016 in the Region of the Americas and discuss the challenges and opportunities for achieving the proposed target of 10% relative reduction by 2030.

## Estimating annual per capita consumption

APC has considerable measurement error but is considered the most accurate and precise indicator of alcohol exposure (8, 9). The total per capita alcohol consumption is the sum of recorded and unrecorded consumption, adjusted for tourism alcohol consumption (2). The World Health Organization (WHO) uses several data sources to estimate APC. That includes data from the WHO Global Survey on Alcohol and Health, completed by focal points appointed by the ministries of health of Member States, as well as data from the alcohol industry (publicly available sources), the Food and Agriculture Organization of the United Nations (FAO), and other sources (2).

## Recorded alcohol consumption

Estimating the average volume of recorded alcohol consumption in a country is best done using national sales, production, and/or taxation data, as population surveys most often underestimate total alcohol consumption when compared with these national data (8). Retail sales data are the most accurate means of estimating how alcohol is consumed in a population, as governments often monitor sales data for tax collection purposes (8). Although there are some limitations to this type of data (e.g., beverages can be purchased yet not consumed in the same year, stockpiling can occur before a tax increase, neither home production nor smuggling are accounted for), it is still a relatively reliable source. Additionally, sales, production, and taxation data systematically exclude some sources of alcohol that account for a fair portion of the total alcohol consumed in a country; these sources of alcohol are known as unrecorded sources. Furthermore, sales, production, and taxation data do not account for alcohol that is consumed by tourists while they are visiting a country (8).

The four major categories of alcoholic beverages (beer, wine, distilled spirits, and all other beverages) available within a country should be included in consumption estimates. However, in many developing countries the alcohol beverage market is made up of local beverages, such as cider, fruit wines, chicha, shochu, aguardiente, cachaça, and samsu, which may be as important as beer, wine, and distilled spirits (8). Furthermore, additional survey data can provide information on who drinks what types of beverages (at least by gender and age groups), which can then be useful in monitoring trends in consumption and relating specific beverages to specific harms. For standardization purposes, APC figures are given in liters of pure alcohol, thereby requiring estimates and/or assumptions about the alcohol content of different beverages. Beer, for example, is usually estimated at 5% pure alcohol (the most-sold alcohol per volume type of beer), but this can vary widely, ranging from 0.9% up to 12% or above (8).

## Unrecorded alcohol consumption

The WHO *Global Status Report on Alcohol and Health 2018* estimated unrecorded alcohol consumption as a percentage of total alcohol consumption (2). A regression analysis was used to estimate country-level proportions of unrecorded alcohol consumption. Estimates of unrecorded alcohol consumption were obtained from four sources: 1) Expert judgements from a WHO survey of experts based on whether any changes in unrecorded consumption had occurred since 2010 (i.e., since the 2014 WHO *Global Status Report on Alcohol and Health*), the magnitude of these changes, and documented supporting evidence; 2) a WHO and Centre for Addiction and Mental Health (CAMH) nominal expert group Delphi survey in 2013; 3) a second WHO and CAMH nominal expert group Delphi survey in 2018; and 4) WHO’s STEPwise approach to surveillance (STEPS) surveys. The percentage of unrecorded alcohol consumption to total consumption was estimated via a regression model based on these input data (2).

## Tourist alcohol consumption

Data for tourist estimations are obtained from the Institute for Health Metrics and Evaluation (IHME), which is part of the WHO Global Information System on Alcohol and Health (GISAH) (2). The liters of alcohol consumed by tourists in a country was established based on the number of tourists who visited a country, the average amount of time spent in the country, and how much these individuals drink on average in their countries of origin. In addition, tourist alcohol consumption measurements also take into consideration the people of the country who are consuming alcohol while visiting other countries. The estimations take into account the following assumptions: 1) that people drink the same amounts of alcohol when they are tourists as they do in their home countries; and 2) that tourist consumption globally is equal to zero and thus can either be net negative or positive at country level (2). However, for those countries with a small population and a large volume of tourism, tourist consumption may impact APC measurements, and if not taken into account, this may distort estimates of alcohol consumption and the alcohol-attributable burden of disease and injury (3).

## MATERIALS AND METHODS

Data from WHO GISAH were used to calculate the annual percent change of APC in 35 countries in the Americas. Three subgroup analyses were defined: data from 1990–2009, 2010–2016, and 1990–2016, aiming to identify different trends in annual percent change of APC before and after the adoption of the WHO Global Strategy to Reduce the Harmful Use of Alcohol (10). For the analysis presented in the figures, a time series was built based on the liters of pure ethanol per capita estimated for each country. The base 10 logarithmic transformation of the annual percent change of APC for total population and among drinkers, in liters of pure ethanol, percentage was considered as a dependent variable (y) and the centralized year as an independent variable (x). The Prais–Winsten model was used for trend analysis. A trend was present when zero did not fall within the 95% confidence interval (CI), where (a) trend was of an increase when annual percent change was positive and (b) trend was of a decrease when the annual percent change was negative. When zero fell within the annual percent change of APC 95% CI, the trend was considered to be “no change.”

The annual percent change of APC of drinkers only was also analyzed for the same time period. Drinkers were defined as people over 15 years of age who had at least one standard drink in the last 12 months (2). The estimated number of drinkers for the population of each country was based on information from national surveys on alcohol consumption (2).

## RESULTS

**FIGURE 1.**
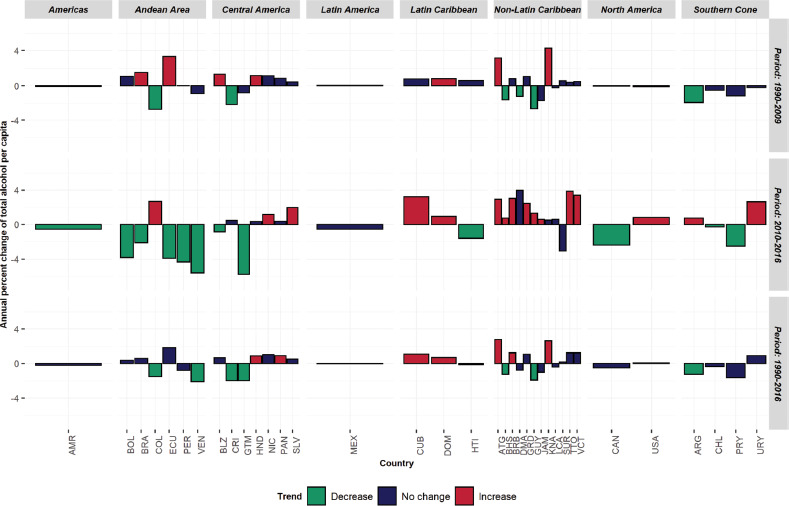
Annual percent change of total alcohol per capita consumption in 35 countries in the Americas during three periods of time (1990–2016; 1990–2009; 2010–2016)

***Source*:** Prepared by the authors based on data from https://apps.who.int/gho/data/node.main.GISAH

In total, 35 countries of the Americas were included in the analysis. Figure 1 shows that the Americas as a whole showed a trend of no change in annual percent change of total APC for the total period, with a slight decrease in the period 2010–2016.

Over the whole period, 1990–2016, seven countries showed a slight increase in annual percent change of APC, and seven countries showed a slight decrease. It should be noted that that the annual percent change of total APC was found to be less than 6% in either increase or decrease throughout each of the time periods examined.

In contrast, over the 2010–2016 period, 11 countries showed a decrease overall in the annual percent change of total APC, while 16 countries showed an increase. The largest increases were found in the recent years in Cuba, Colombia, Uruguay, El Salvador, and several countries of the Non-Latin Caribbean. The largest decreases were reported in Venezuela and Guatemala.

**FIGURE 2.**
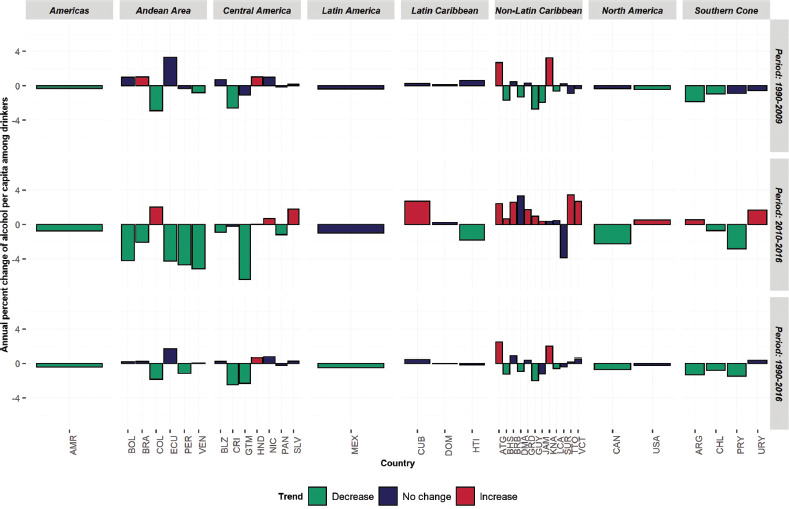
Annual percent change of alcohol per capita among drinkers in 35 countries in the Americas during three periods of time (1990–2016; 1990–2009; 2010–2016)

***Source*:** Prepared by the authors based on data from https://apps.who.int/gho/data/node.main.GISAH

When comparing the two periods of trend, before and after the adoption of the Global Alcohol Strategy in 2010, the scenario is significantly different in some countries. In Colombia and Brazil, the trend of increase until 2009 was reversed for a trend of reduction from 2010, resulting in a trend of no change in the total period. On the other hand, countries like Dominica and Antigua and Barbuda showed a trend of increase in both periods, but no country showed a trend of decrease in both periods.

The results show that in the general population, when considering the total period (1990–2016), all the countries that presented a trend of annual increase were located in the Latin Caribbean, Non-Latin Caribbean, and Central America, including Honduras, Panama, Cuba, Dominican Republic, Antigua and Barbuda, Barbados, and Saint Kitts and Nevis.

Figure 2 shows the same analysis of annual percent change of total APC in the three time periods but restricted to current drinkers. The trends are broadly similar to the ones described above. However, some statistical differences were found. For example, a trend of no change for the total sample in the total period in the Americas was a trend of decrease when only current drinkers were looked at. The same occurred in Canada and Mexico. However, the opposite was found in Panama over the same time period.

Over the whole time period, the trends showed that among drinkers, annual percent change of total APC increased in Honduras, Antigua and Barbuda, and Saint Kitts and Nevis. These increases were all less than 4%. Annual percent change of total APC for drinkers decreased over the whole time period in Colombia, Peru, Costa Rica, Guatemala, Mexico, the Bahamas, Dominica, Guyana, and Saint Lucia. All of these decreases were of 2% or less.

## DISCUSSION

As a whole, the Americas showed no change in annual percent change of total APC during the period 1990–2016, with a slight decrease in the period 2010–2016. The trends for current drinkers were broadly the same. This is consistent with the consensus that APC is an adequate indicator of the harmful use of alcohol (9).

There were, however, some differences across the region when considering trends of annual increase in annual percent change of total APC. Of the 11 countries that showed a decrease during the period 2010–2016, only Ecuador adjusts its excise tax for inflation (3), while Canada, at the subnational level, had implemented minimum alcohol pricing policies for on-premise sales in 9 out of 13 provinces and territories, and 5 out of 13 had these policies for off-premise sales (11). Adjusting excise taxes to inflation and minimum pricing policies are considered very cost-effective policies to reduce harmful alcohol use.

However, this analysis does not demonstrate causal links, but it is possible that these policy changes played a role in the modest decrease in percentage in APC over this period. At the same time, excise taxes also need to lead to a reduced affordability to have an impact on consumption, which is more difficult to know; countries that have such taxes have not assessed the impact on alcohol demand. It may also be speculated that for countries like Venezuela, Haiti, Guatemala, and Bolivia, the higher levels of poverty (12) in these countries may have impacted the affordability of alcohol, therefore decreasing the annual percent change of APC when no alcohol policy was implemented.

The lack of change in APC throughout the region suggests that the adoption of the WHO Global Alcohol Strategy in 2010 has not resulted in the implementation of effective alcohol policies. The SDG target of a relative decrease of 10% in the harmful use of alcohol by 2030 (13) may not be met if current trends continue. Unless effective policy measures to reduce APC are put into place as soon as possible, the significant impact of alcohol on mortality, morbidity, and development will not be reduced, and may actually increase (14).

A further challenge faced in the effort to meet SDG 3.5 is the difficulty of collecting data on APC across the region. Only Argentina, Canada, Chile, United States of America, and Uruguay provide government data to WHO. For the other countries in the region, the data used are a mix of industry sources and data from FAO (2).

The main limitation of this study is the quality of the data on which it is based. As mentioned above, currently, estimates from WHO for the majority of countries in the Americas come from alcohol industry sources, which cannot be independently validated, or the FAO (2). In addition, unrecorded alcohol consumption is not estimated at country level. This involves several types of alcohol products, some illegal, which pose challenges to their systematic assessment.

### What can be done to reduce alcohol per capita consumption?

The most cost-effective policies to reduce APC are those related to the control of alcohol availability to the entire population (15). These fall broadly into three categories: 1) alcohol affordability (through pricing and taxation policies); 2) alcohol physical availability (through controls on hours and days of alcohol sales, density of outlets, and age limits to purchase and drink alcohol); and 3) alcohol marketing, including advertising, promotions, and sponsorships (through an effect on the age of initiation and excessive drinking among children and adolescents) (15). These policies, commonly referred to as the “best buys,” are recommended by WHO and based on extensive scientific evidence. These are part of the recommended policies aimed at preventing and reducing the burden of noncommunicable diseases globally and in the Region of the Americas (15). A recent regional analysis of alcohol policies implemented in all countries of the Region, conducted in 2020, based on a series of composite scores, indicated that the lowest scores were obtained for the “best buys” policies, including pricing policies and marketing of alcoholic beverages (16).

### Conclusion

Alcohol is a significant obstacle to sustainable human development across health, economic, social, and environmental areas (13). Given that the findings from this study show no significant change on APC across the region over the last 25 years, it can be concluded that the current policy response has been inadequate. Countries in the Americas have made little progress toward preventing alcohol use and related harms, as well as protecting vulnerable groups from the harms of alcohol consumption (16). Unless action is taken, it is likely that the harmful use of alcohol will increase (2). Increased regulation of alcohol through alcohol excise taxes, comprehensive marketing restrictions, and limits on the hours of retail and alcohol sales need to be considered essential public health tools for reducing APC and harms from alcohol (15).

Though data are available in most countries, they are not used or accessed by those in charge of reporting on the SDGs and in health surveillance. The Pan American Health Organization (PAHO) is advancing efforts to build country capacity to do that through the development of tools and training of country teams (16). PAHO is also working in technical cooperation activities at the country level to promote the implementation of national alcohol plans and effective policies.

To help promote the “best buys” and other effective policies, WHO launched the SAFER initiative in 2018 (17). The WHO SAFER initiative was launched with the aim of helping countries to implement guidelines and instruments related to alcohol harm prevention. SAFER is an acronym for five cost-effective interventions to reduce alcohol harm (18).^1^ SAFER is based on the accumulated evidence of cost-effectiveness of different alcohol control measures. It recognizes the need to protect public health oriented policy-making from alcohol industry interference as well as to have strong monitoring systems to ensure accountability and track progress in its implementation (14). PAHO is currently working in Argentina, Bolivia, and Mexico on the development of road maps to promote the implementation of the SAFER initiative.

## Disclaimer.

Authors hold sole responsibility for the views expressed in the manuscript, which may not necessarily reflect the opinion or policy of the *Revista Panamericana de Salud Pública/Pan American Journal of Public Health* and/or those of the Pan American Health Organization.

## Footnotes

1 **SAFER: S**trengthen restrictions on alcohol availability; **A**dvance and enforce drink-driving countermeasures; **F**acilitate access to screening, brief interventions, and treatment; **E**nforce bans or comprehensive restrictions on alcohol advertising, sponsorship, and promotion; **R**aise prices on alcohol through excise taxes and pricing policies.
